# Sudden Death Due to Rupture of Ectopic Pregnancy in a Young Lactating Mother

**DOI:** 10.7759/cureus.33598

**Published:** 2023-01-10

**Authors:** Atul S Keche, Poovaragavan V, Sravan JS

**Affiliations:** 1 Forensic Medicine, All India Institute of Medical Sciences (AIIMS) Bhopal, Bhopal, IND; 2 Forensic Medicine and Toxicology, Manipal Tata Medical College, Jamshedpur, IND

**Keywords:** young mother, lactational amenorrhoea, reproductive age, sudden death, ruptured ectopic pregnancy

## Abstract

The incidence of ectopic pregnancy in India is increasing over time along with a considerable mortality rate. The common site for ectopic pregnancy is the fallopian tubes and is also associated with a higher incidence of mortality following the tubal rupture. Even though lactational amenorrhea prevents pregnancy to some extent, the probability of pregnancy increases when the frequency of lactation decreases. Rupture of ectopic pregnancy can occur abruptly without any serious symptoms as well. One should not ignore even mild symptoms in females of reproductive age group especially those who are sexually active.

A 25-year-old female with an alleged history of sudden onset of severe abdominal pain was taken to AIIMS Bhopal, where she was declared brought dead and her body was sent for autopsy. She got married at the age of 23 years and was still breastfeeding her 11-month-old child.

The autopsy revealed 2.2 liters of fluid and clotted blood in the peritoneal cavity. The right fallopian tube enlarged along with a tear of size 1cm x 1cm evident on the anterior aspect. Meticulous dissection revealed a membranous sac of size 4cm x 2cm x 2cm containing a placenta of size 1cm x 1cm and an embryo of size 2cm x 1cm. All other organs were pale and normal. The uterine cavity was found empty. The cause of death was attributed to ruptured ectopic pregnancy.

## Introduction

The process of normal pregnancy starts with the fertilization of an egg by sperm followed by implantation of the fertilized egg in the uterine cavity. When a fertilized ovum gets implanted and develops outside the uterus, it is known as ectopic pregnancy. The most common site of ectopic pregnancy is the fallopian tubes, the other sites are the cervix, ovary, and abdominal cavity [1]. Ectopic pregnancy is reported in one in every 100 to 150 pregnancies with major risk factors being age, infertility, smoking, previous ectopic pregnancy, intrauterine device usage, pelvic inflammatory diseases, etc. [2].

The suspicion of ectopic pregnancy arises when a woman presented with a history of abdominal pain and vaginal bleeding with a positive pregnancy test. Prompt diagnosis and timely management can be lifesaving. Unattended tubal pregnancy can rupture and ultimately result in fatality [3].

Lactational amenorrhea or postpartum infertility means temporary postpartum infertility, that occurs when the woman is amenorrhoeic and actively breastfeeding. A strong relationship has been found between the amount of suckling and the contraceptive effect, such that exclusive breastfeeding along with feeding on demand helps in lengthening the period of effective contraception [4].

## Case presentation

The case

A 25-year-old female, married for two years, was a mother of an 11-month-old male child. She had sudden onset of abdominal discomfort with no history of trauma the previous day evening and brought some over-the-counter medicine and got some temporary relief. The pain got severe at the midnight and she was taken to All India Institute of Medical Sciences (AIIMS), Bhopal where she was declared brought dead and the body was sent for autopsy. She was still breastfeeding her 11-month-old child.

Autopsy

On the external examination, the body was pale and old healed linear lower segment cesarean section scar mark of size 11cm, present horizontally over the lower abdomen just above the pubic symphysis.

**Figure 1:**
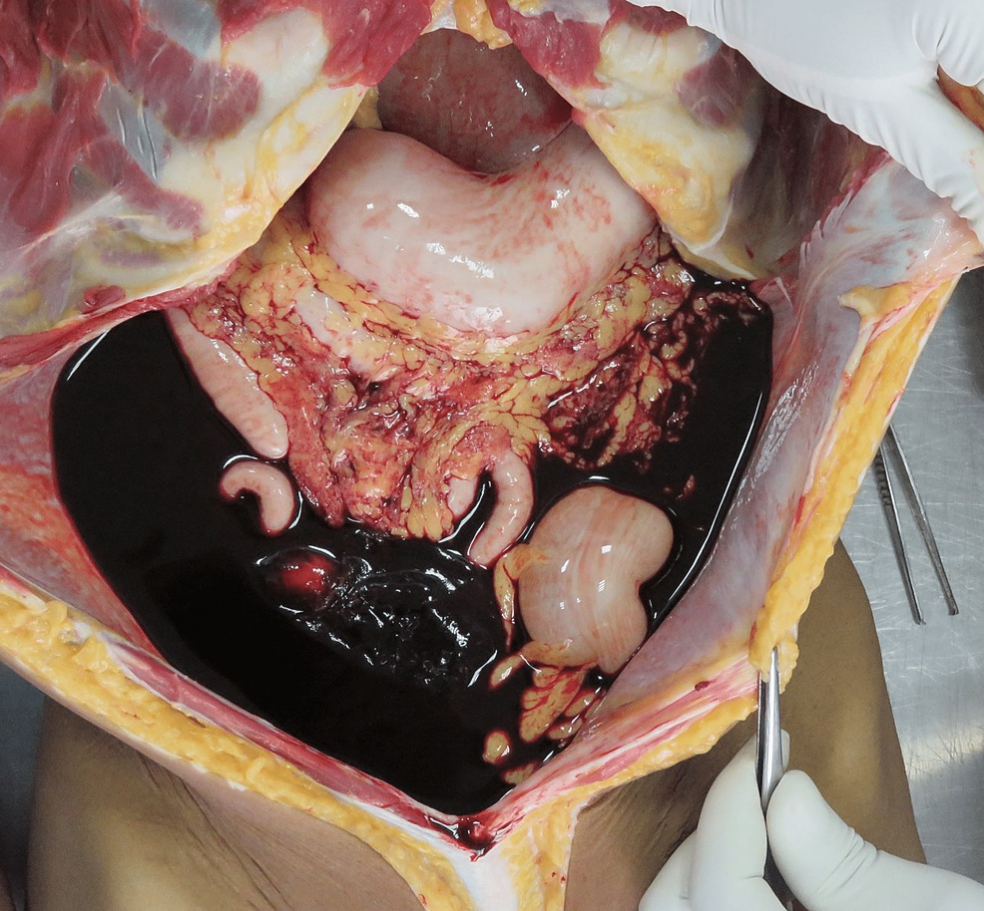
Peritoneal cavity filled with blood

**Figure 2:**
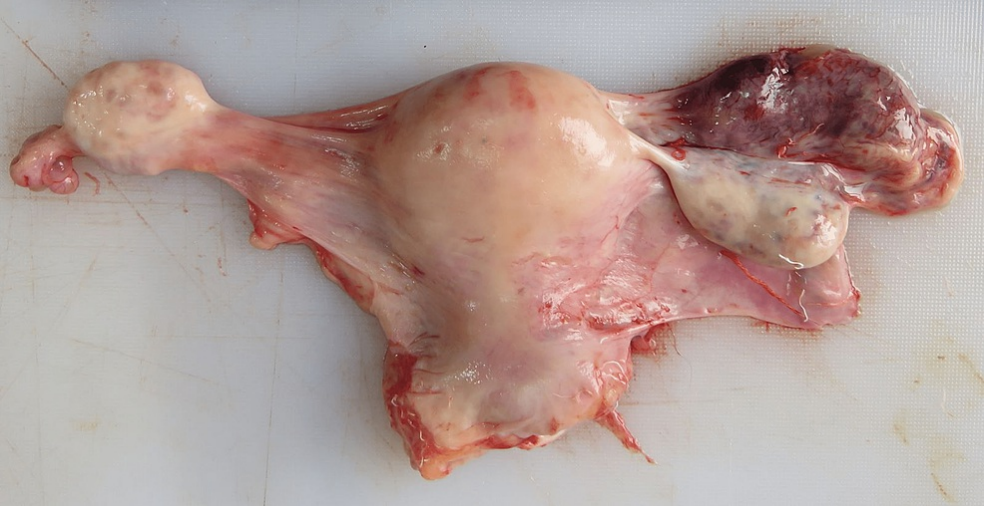
Uterus with swelling evident in the right fallopian tube

**Figure 3:**
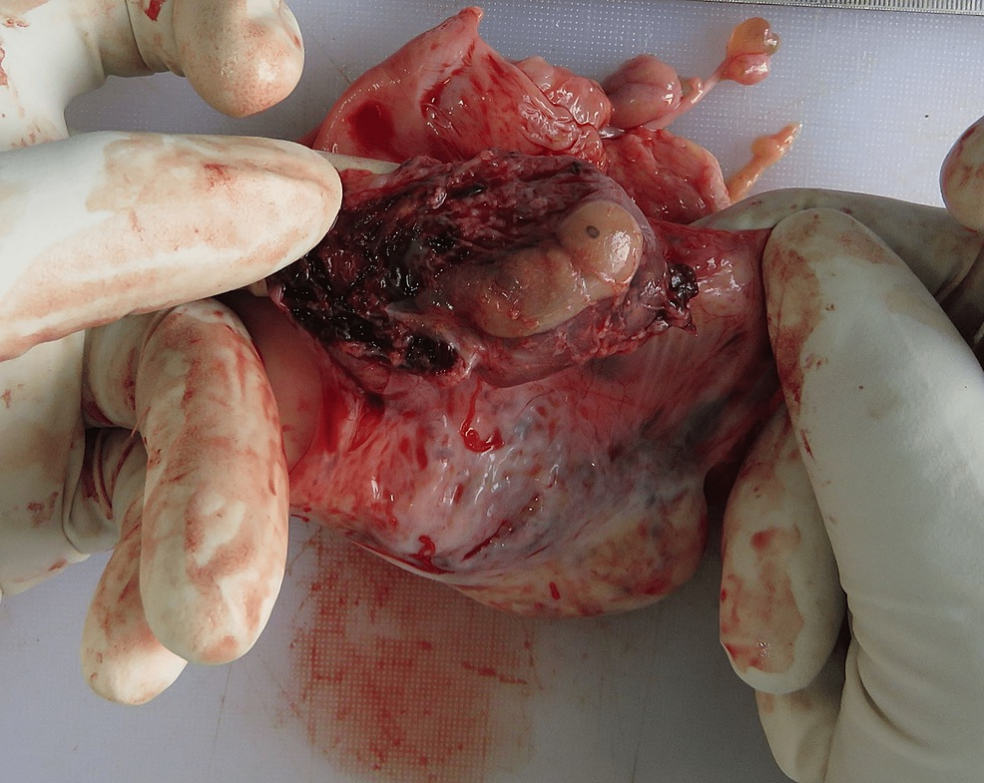
Fallopian tube contains a membranous sac

**Figure 4:**
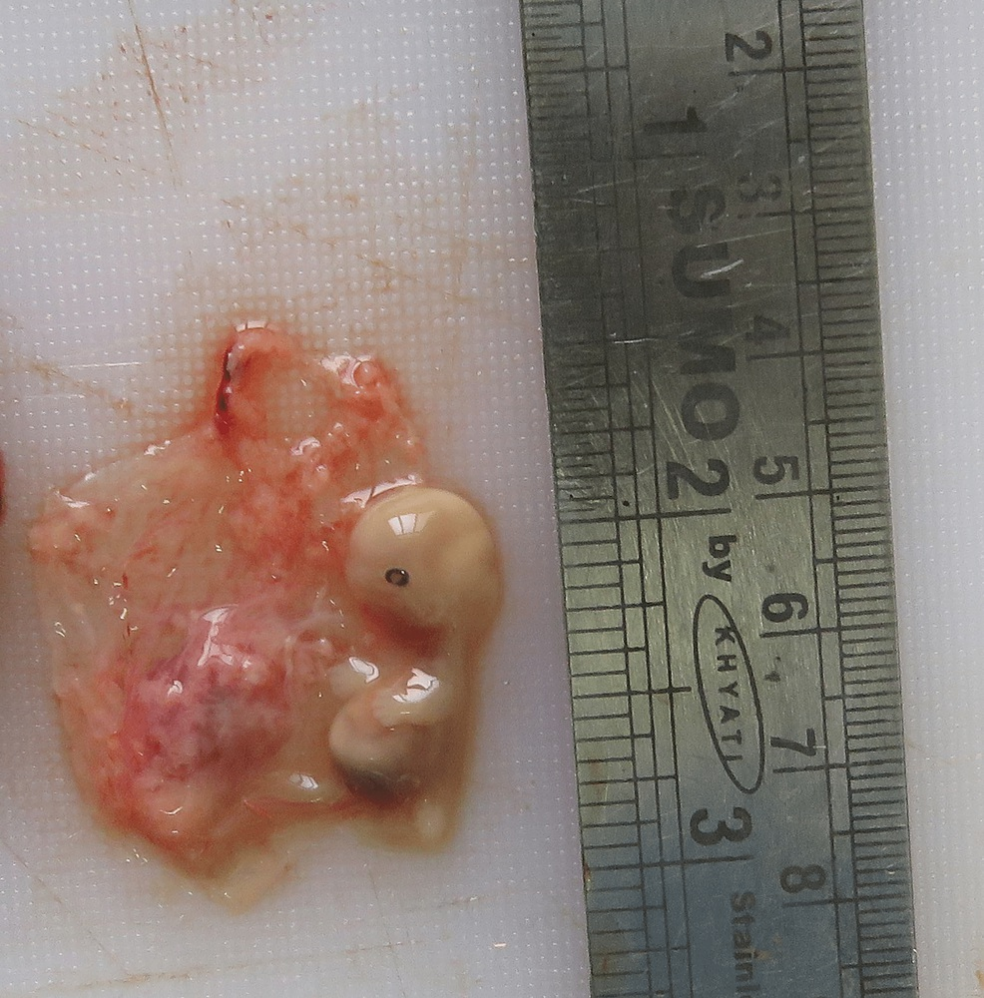
Membranous sac contains an embryo and placenta

On internal examination, around 2.2 liters of fluid and clotted blood were present in the peritoneal cavity (Figure 1). The blood was collected by soaking the blood in a gauze piece and the difference in weight between the unsoaked and soaked gauze pieces was noted to get the blood volume. Swelling of the right fallopian tube along with rupture of size 1 x 1cm was evident (Figure 2). The fallopian tube on the right side was thinned out with ecchymosis evident. On meticulous dissection, a membranous sac of size 4 x 2 x 2cm was evident (Figure 3), containing a placenta of size 1 x 1cm and an embryo of size 2 x 1cm. The embryo had eyes seen as two black spots but the mouth cleft and limb buds were not appreciable (Figure 4). The left fallopian tube was normal. Weight of uterus and adnexa: 114 grams. The uterine cavity was found empty.

All other organs were intact and pale. The stomach contained about 100ml of partially digested food particles without any smell and had normal mucosa. Uterus with cervix measured 8cm x 5.5cm x 3.5cm. The cause of death was attributed to hemorrhage and shock as a result of the rupture of an ectopic tubal pregnancy.

## Discussion

Ectopic pregnancy is the most common cause of maternal death in the first trimester of pregnancy. The fallopian tube is the most common site for ectopic pregnancy and is associated with a higher incidence of mortality following tubal rupture. The ampulla is the commonest site in the fallopian tube. The incidence of rupture ectopic pregnancy has been increasing over the years [3].

There are a lot of risk factors causing tubal damage and dysfunction. Risk factors are tubal sterilization, history of previous ectopic pregnancy, any other pathology involving the fallopian tubes, genital or pelvic infection, multiple sexual partners, pelvic or abdominal surgery, smoking, abortion at younger ages, etc. The classic signs of ectopic pregnancy are abdominal pain, amenorrhea, and vaginal bleeding. Patients presenting with severe abdominal pain, tenderness, and shock indicate ruptured ectopic pregnancy [5]. Early diagnosis of tubal pregnancy can reduce maternal mortality and morbidity.

The lactational amenorrhea method (LAM) is one of the accepted and effective methods of family planning as per World Health Organization [6]. Also, studies showed that, when used as per recommendation, LAM can be effective as 98% of contraceptive pills and other modern methods. But the duration of lactational amenorrhoea (up to six months or even longer in some cases) and its role as a family planning method in developing countries is not clear [7]. Breastfeeding, by interrupting the pulsatile release pattern of gonadotropin-releasing hormone (GnRH) from the hypothalamus and then the luteinizing hormone (LH) from the pituitary, thereby delays the resumption of normal ovarian cycles. Lactation only interrupts the ovarian cycle; it does not completely withhold the occurrence of the ovulation cycle. The period of return of menstruation following childbirth varies from person to person and the return does not mean that a woman has started her ovulation again [8].

In this case, the mother was still breastfeeding but the frequency of lactation was minimal along with failure to take any other contraceptive measures as a barrier method resulting in pregnancy. Many women are not aware fully of the concept of LAM, they misinterpret it like they won’t get pregnant as long as they are breastfeeding. Even though lactational amenorrhea prevents pregnancy to some extent, the probability of pregnancy increases when the frequency of lactation decreases [9]. This is true in this case as well.

Rupture of ectopic pregnancy can occur abruptly without any serious symptoms [10]. In this case, the deceased had mild abdominal discomfort at first but she ignored it thinking it was some gastrointestinal upset due to food, but the pain worsened over time and ultimately resulted in death before she reached the hospital. So, one should not ignore even mild symptoms in females of the reproductive age group especially those who are sexually active.

## Conclusions

Pelvic inflammatory disease, anatomical defect, conception despite tubal ligation and intrauterine device, and pathology of the fallopian tube are the main causes of ectopic pregnancy. Also, nowadays due to various infertility treatment methods and a lack of interest in exclusive breastfeeding in many women, the incidence rate of ectopic pregnancy is escalating. Lactational amenorrhea is an effective method of contraception when used as per guidelines, but it has its own limitations as well. Immediate reporting to the doctor, early diagnosis, and quick surgical intervention are essential to prevent these kinds of deaths.
